# TLR signaling inhibitor, phenylmethimazole, in combination with tamoxifen inhibits human breast cancer cell viability and migration

**DOI:** 10.18632/oncotarget.10358

**Published:** 2016-07-01

**Authors:** Anthony L. Schwartz, Eric Dickerson, Nilesh Dagia, Ramiro Malgor, Kelly D. McCall

**Affiliations:** ^1^ Athencion Biotechnology Corporation, Suite 100, Marietta, OH 45750, USA; ^2^ Department of Biomedical Sciences, Appalachian Rural Health Institute, Diabetes Research Center, College of Osteopathic Medicine, Ohio University, Athens, OH 45701, USA; ^3^ Oxygen Healthcare Research Pvt. Ltd, Ahmedabad, Gujarat 382213, India; ^4^ Department of Specialty Medicine, Appalachian Rural Health Institute, Diabetes Research Center, College of Osteopathic Medicine, Ohio University, Athens, OH 45701, USA

**Keywords:** breast cancer, toll-like receptors, cytokines, tamoxifen, chemotherapy

## Abstract

Heightened co-expression and dysregulated signaling associated with Toll-like receptor 3 (TLR3) and Wnt5a is an integral component of solid tumors and hematological malignancies. Our previous findings in pancreatic cancer and melanoma suggest that inhibition of these pathways by a TLR3 signaling inhibitor, phenylmethimazole (C10), results in significantly decreased IL-6 levels, STAT3 phosphorylation, minimal cancer cell migration and reduced cancer cell growth *in vitro* and *in vivo*. In this study, we extended our earlier observations by performing studies in human breast cancer cells. We found that human MCF-7 breast cancer cells express high basal levels of TLR3 and Wnt5a RNA. C10 treatment resulted in significantly decreased TLR3 and Wnt5a expression levels. This functionally translated into significantly reduced IL-6 levels and STAT3 phosphorylation *in vitro*. In addition, the inhibition of this signaling cascade by C10 further resulted in decreased cell viability and migration of MCF-7 cells. Strikingly, the combination of C10 and tamoxifen, the standard of care therapy for breast cancer, further decrease cancer cell growth better than either agent alone. These data support the novel finding that inhibition of TLR3 signaling in combination with tamoxifen, may increase the effectiveness of current treatments of breast cancer.

## INTRODUCTION

Extensive research from *in vitro*, *in vivo* and clinical studies has now established that Toll-like receptors (TLRs) are important in the development of carcinogenesis and tumor progression of a variety of cancers. Indeed, heightened expression of TLRs is evidenced in multiple cancer cell lines, and in tumor cells and tissues obtained from patients [1–3]. Furthermore, activation of TLRs on cancer cells promotes chronic inflammation which stimulates cancer cell proliferation, migration, tumor angiogenesis and creates a tumor microenvironment which impairs the anti-tumor function of the immune system allowing tumors to develop and survive [4].

Much like TLRs, the Wnt family proteins have also been implicated in carcinogenesis - in both the initiation and maintenance of cancer [5–7]. *In vitro* studies with various cancer cell lines and tumor specimens from patients have revealed elevated expression of Wnt proteins (e.g., Wnt5a) and deregulated Wnt signal transduction pathways [5, 8–11]. The elevated expression of Wnt proteins (e.g., Wnt5a) in tumor samples correlates with advanced stages and poor prognosis in solid tumors as well as hematological malignancies [5, 8–11].

The TLR-mediated pro-inflammatory cytokine, IL-6, is another integral molecular effector of initiation and growth of malignant tumors. This pro-inflammatory cytokine is implicated in a milieu of cancer types because of its propensity to drive the activation of the oncogenic transcription factor signal transducer and activator of transcription 3 (STAT3). By virtue of interacting with other transcription factors (e.g., NF-B), STAT3 is known to mediate crosstalk between tumor cells and inflammatory cells within the tumor microenvironment and promote the development and progression of multiple types of human cancers [12–17]. The expression of STAT3 and presence of its active form (i.e., phosphorylated form) is increased in tumor tissues from patients and this pronounced expression indicates a poor prognosis in a variety of cancers [18].

Although much fundamental, translational and clinical research has been conducted on TLRs, Wnts and IL-6/STAT3, the majority of the investigations have studied these cardinal mediators of oncogenesis in “isolation”. Only recently has there has been an increasing interest in “co-associating” and tying these aforementioned oncologic mediators together. An IL-6/STAT3/Wnt5a signaling loop has been described by different groups [19, 20]. Our group demonstrated that IL-6, a TLR signaling product, can activate STAT3 with resulting overexpression of Wnt5a in human papillary thyroid carcinoma, melanoma and pancreatic cancer [1, 2]. Importantly, a novel inhibitor of pathologic TLR3 signaling, phenylmethimazole (C10), has the ability to block IL-6 production, as well as decrease viability/growth and migration in these same cancers [2, 20]. We hypothesized that the effect of C10 on viability/growth and migration of these cancers was related to its suppressive effect on TLR3 signaling which led to the inhibition of TLR-mediated STAT3 and Wnt5a signaling [20]. Moreover, in these reports we showed that C10 also inhibited tumor growth in mouse xenograft models of human pancreatic cancer and malignant melanoma thus indicating the potential of using a TLR inhibitor for the treatment of TLR-expressing cancers [2]. In this present study, we sought to extend our earlier observations of TLR3 and Wnt5a association and their role in tumor viability/growth and migration in human breast cancer. We also evaluated the ability of C10 to synergistically inhibit tumor cell viability/growth in combination with tamoxifen, the current standard of care therapy for breast cancer.

## RESULTS

### C10 significantly decreases high basal TLR3 and Wnt5a expression in human breast cancer cells

**Figure 1:**
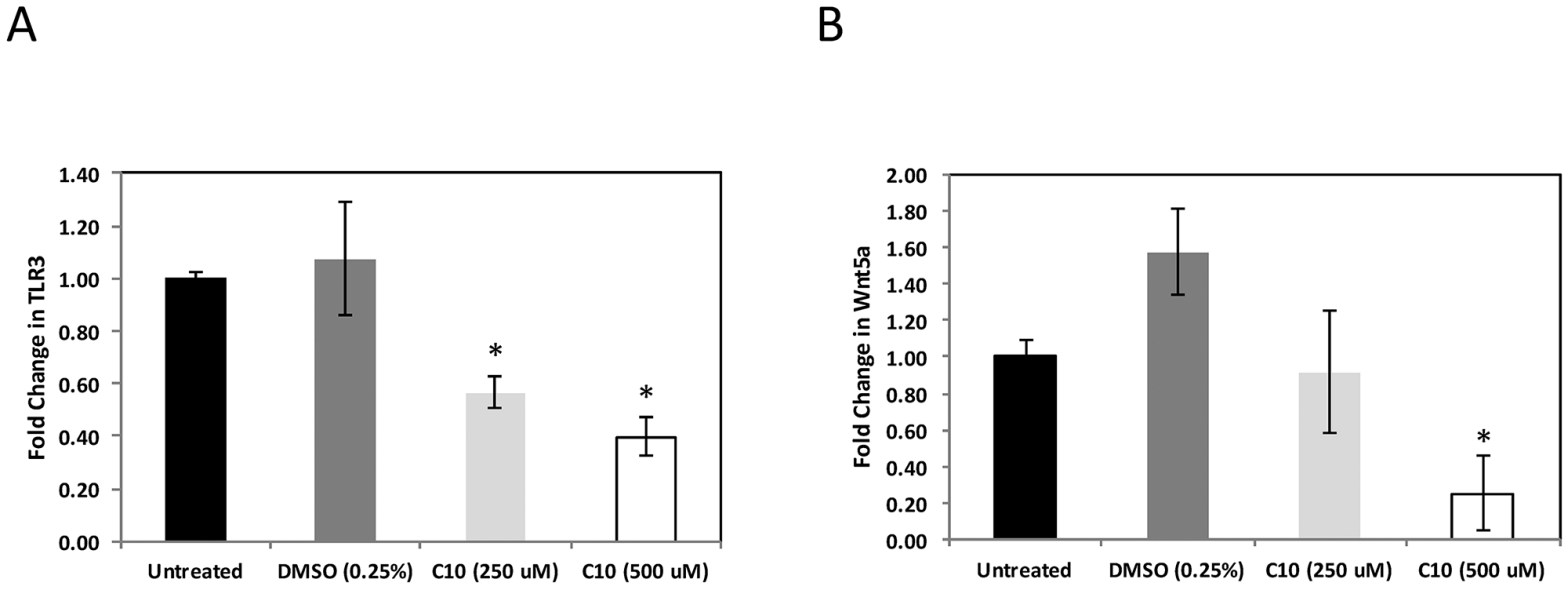
C10 significantly decreases high TLR3 and Wnt5a expression in MCF-7 cells MCF-7 human breast cancer cells were grown to approximately 100% confluency. Subsequently, cells were treated with 250-500 μM C10 or vehicle control (0.25% DMSO). Following 24 hr. incubation, cells were harvested, total RNA was collected and gene-specific qRT-PCR was performed for **A.** TLR3 and **B.** Wnt5a. ^*^Indicates significant difference from untreated and DMSO groups, p < 0.05. No significant differences between untreated and DMSO groups. One-way ANOVA with Tukey-Kramer post-hoc analysis. Results are representative of n = 3 separate experiments.

Previous studies in our laboratory demonstrated that C10 decreased high constitutive TLR3 and Wnt5a expression in human malignant melanoma, papillary thyroid, and pancreatic cancer cell lines [1, 2]. In these studies we sought to evaluate the effect of the same TLR signaling inhibitor, C10, on human MCF-7 breast cancer cells that exhibit high constitutive levels of TLR3 and Wnt5a RNA. As was the case in our previous studies, we observed significant decreases in TLR3 RNA levels following C10 treatment in MCF-7 cells (Figure 1A). Similarly, MCF-7 cells also exhibited significant decreases in Wnt5a levels after C10 treatment (Figure 1B).

### C10 significantly decreases IL-6 protein levels and subsequent STAT3^Y705^ phosphorylation

**Figure 2:**
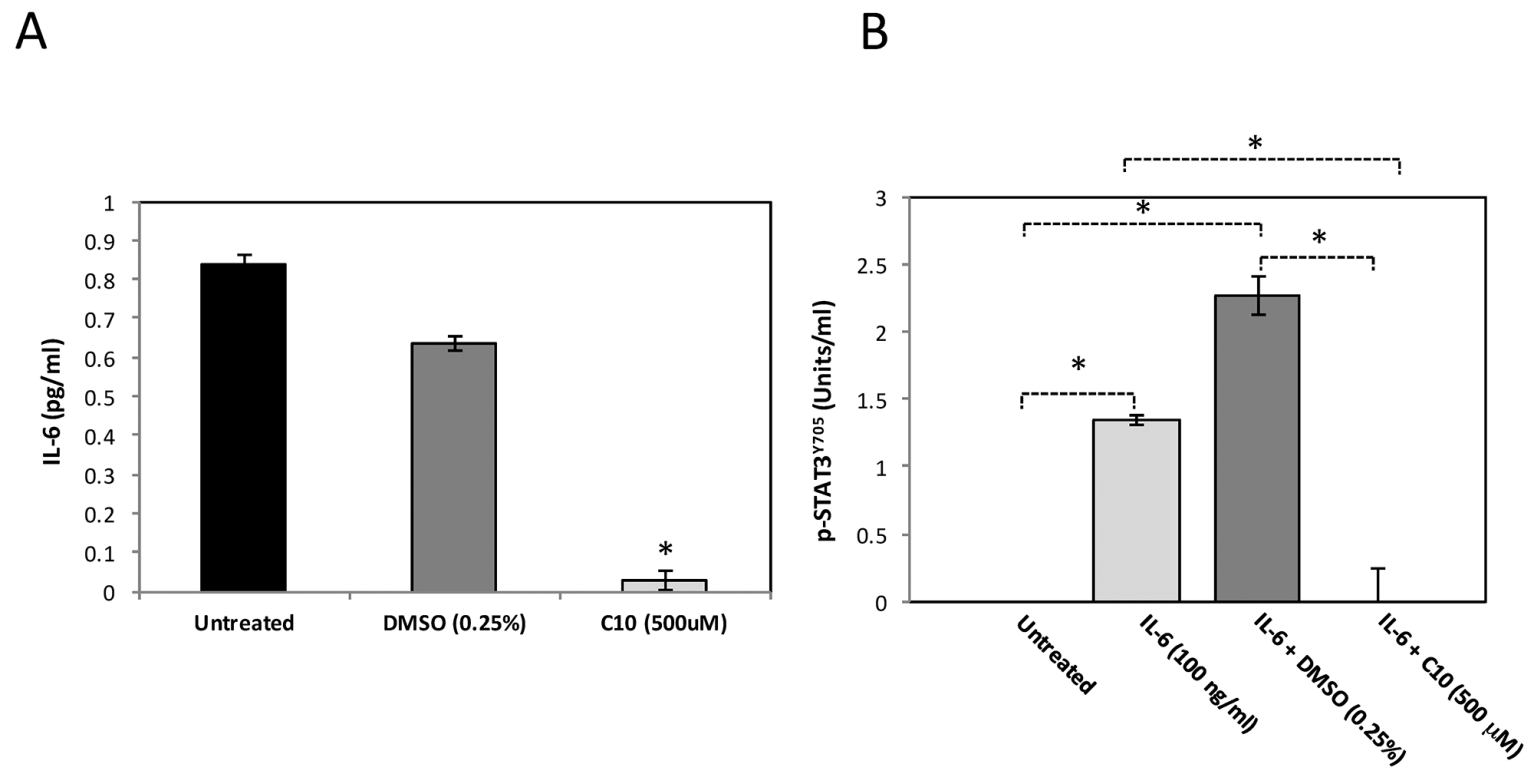
C10 significantly inhibits IL-6 levels and STAT3 phosphorylation in MCF-7 cells **A.** MCF-7 human breast cancer cells were grown to approximately 100% confluency. Cells were then treated with 500 μM C10 or vehicle control (0.25% DMSO). Following a 24 hr. incubation, IL-6 levels were analyzed using ELISA. ^*^Indicates significant difference from untreated and DMSO groups, p < 0.0000001. One-way ANOVA with Tukey-Kramer post-hoc analysis. Results presented are representative of n = 3 separate experiments. **B.** MCF-7 human breast cancer cells were treated with IL-6 for 10 min. to induce STAT3 phosphorylation. In certain cases, MCF-7 cells were pre-treated with vehicle control (0.25% DMSO) or C10 (500 μM) for 1 hr prior to IL-6 stimulation. Subsequently, nuclear protein was extracted and pSTAT3^Y705^ levels were analyzed using ELISA. ^*^Indicates p < 0.0000001. One-way ANOVA with Tukey-Kramer post-hoc analysis. Results presented are representative of n = 3 separate experiments.

IL-6 is a pro-inflammatory cytokine regulated by TLR3 and has been shown to be important for the growth and migration of cancer cells [21–23]. It has also been shown to be an activator of a key oncogene, STAT3, known to mediate uncontrolled cell growth in many types of human cancers [24]. STAT3 is phosphorylated on multiple residues for activation; phosphorylation of both its tyrosine 705 (pSTAT3^Y705^) and serine 727 (pSTAT3^S727^) residues are necessary for maximal transcriptional activity [25–28]. Based on our previous findings that C10 significantly reduced IL-6 protein levels and STAT3 phosphorylated via TLR3 signaling in other cancers, we also evaluated these in MCF-7 breast cancer cells. IL-6 protein levels were analyzed via ELISA. We observed significantly reduced (> 90%) IL-6 protein levels following C10 treatment of MCF-7 cells (Figure 2A). Additionally, we observed a significant decrease in IL-6-induced phosphorylated STAT3^Y705^ in MCF-7 (Figure 2B). Of note, C10 did not affect STAT3^S727^ phosphorylation thus reflecting individual variations of STAT3 phosphorylation among different cancers (data not shown). These data, along with results shown in Figure 1, indicate that the constitutive levels of TLR3 are abnormally high in MCF-7 cells and that downstream signaling (Wnt5a, IL-6 and STAT3) can be inhibited by C10.

### C10 inhibits migration and viability/growth of MCF-7 cells and synergistically enhances the therapeutic effect of tamoxifen in a dose-dependent manner

**Figure 3:**
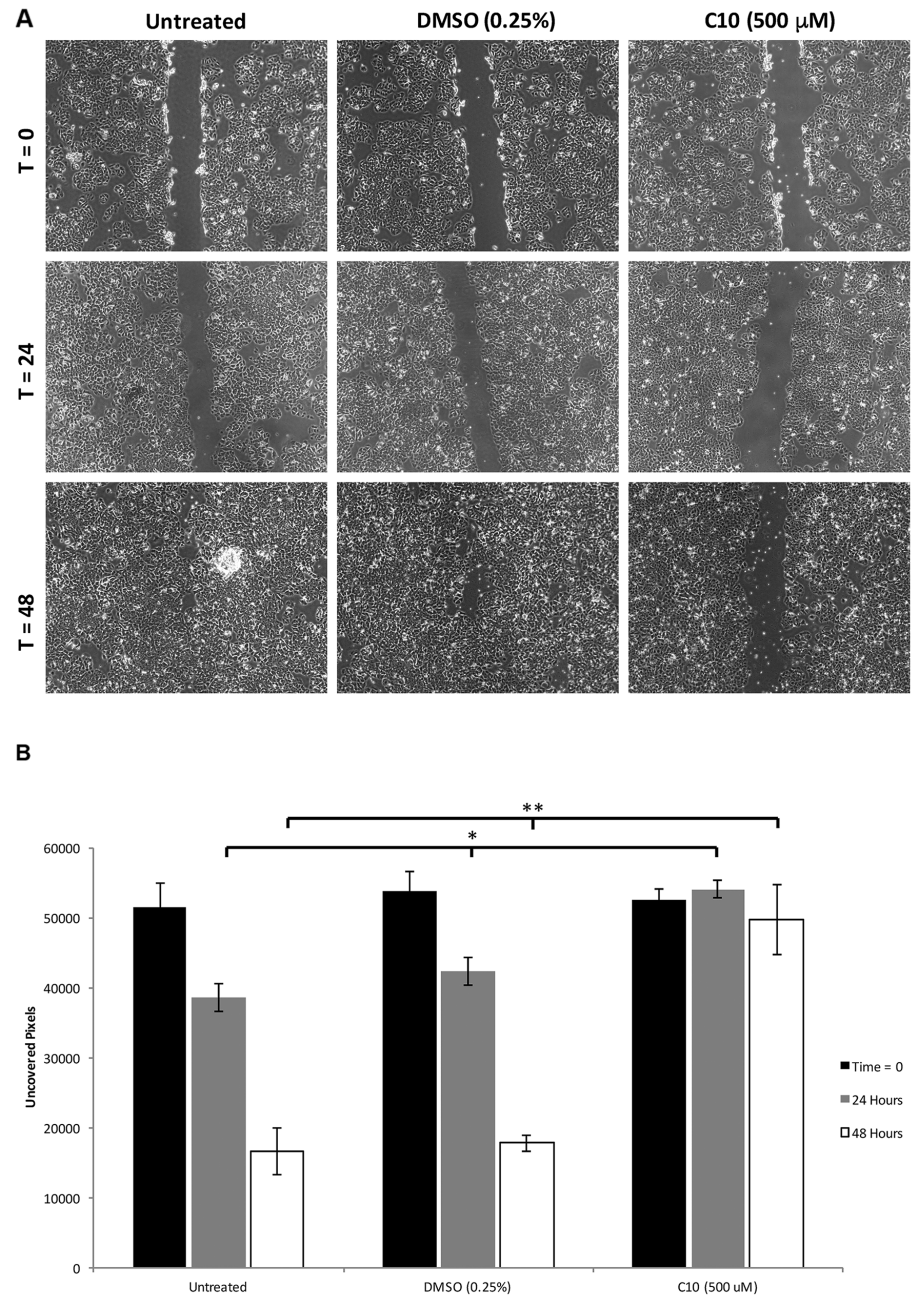
C10 significantly inhibits MCF-7 migration MCF-7 cells were grown to confluency and migration was analyzed using a scratch assay. After the scratch was performed, cells were treated with 500 μM C10 or vehicle control (0.25% DMSO) for 0, 24 and 48 hours. **A.** Representative images of the scratch assay depicting inhibition of migration of MCF7 cells after C10 treatment. **B.** Quantification of cells between each treatment group at 0, 24 and 48 hours. C10 significantly (^*^p < 0.05) inhibits cell migration when compared to DMSO control and untreated groups. Results presented are representative of at least n = 3 separate experiments.

In addition to its effect on constitutive TLR3, Wnt5a levels and subsequent IL-6 production and STAT3 levels/activation, we then evaluated the ability of C10 to inhibit the migration and viability/growth of MCF-7 breast cancer cells. In a scratch assay, cell migration to cover a scratched area on the plate surface containing cells is measured; retention of a visible scratch line as in Figure 3A is evidence of inhibition of cell migration [5]. Treatment of MCF-7 cells with C10 led to significant inhibition of motility/migration as measured using scratch assays at 0, 24 and 48 hours (p < 0.05) (Figure 3A & 3B).

**Figure 4:**
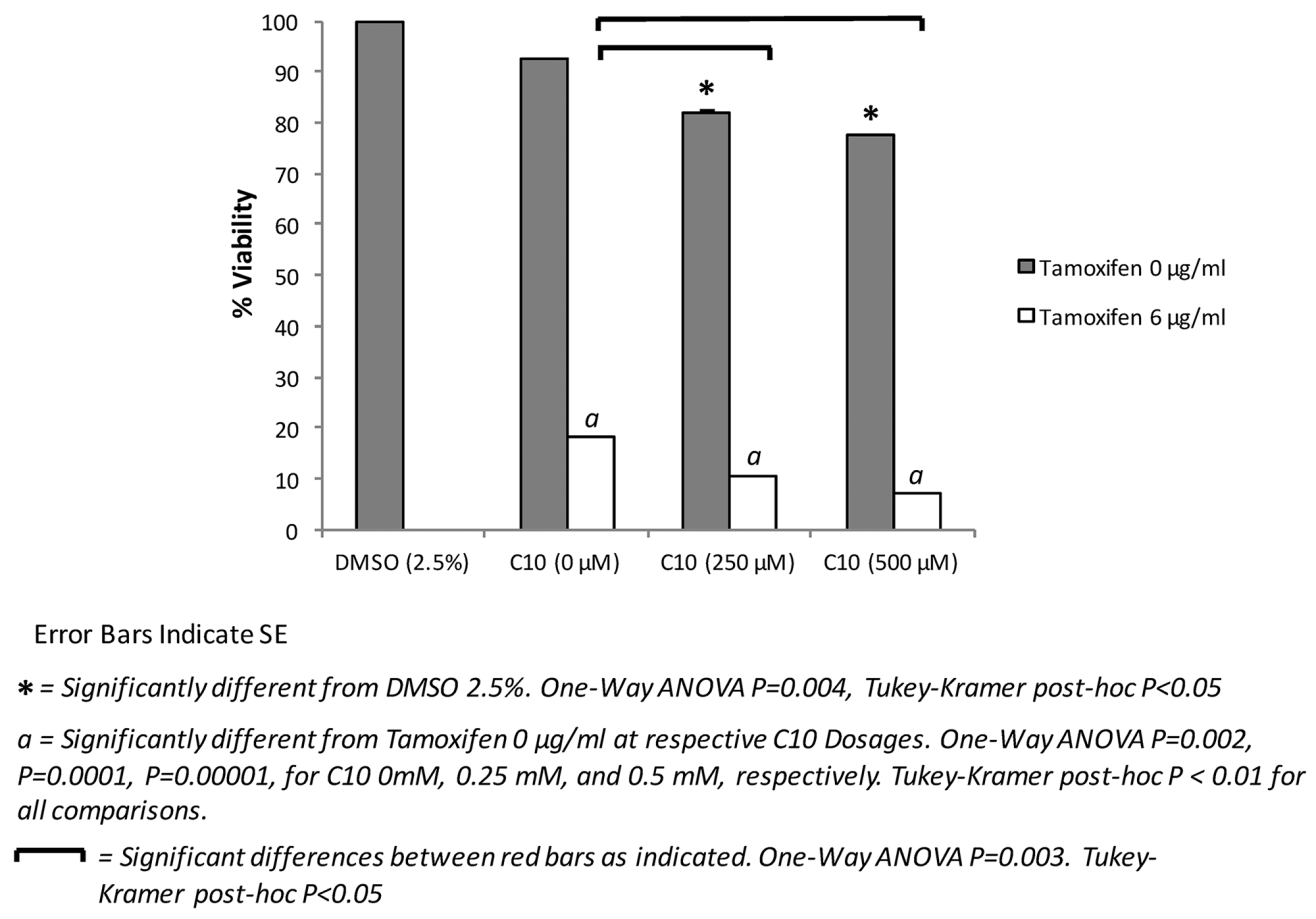
Combination of tamoxifen and C10 significantly inhibits MCF-7 cell viability/growth over Tamoxifen or C10 alone MCF-7 human breast cancer cells were plated onto a 96-well plate, for 72 hrs and then were treated with C10 (0, 250, or 500 μM), vehicle control (0.25% DMSO), tamoxifen (6 μg/ml) or a combination thereof. A MTT-based cell viability assay was then performed to assess cellular viability/growth at 72 hours. C10 further decreases cellular viability/growth in combination with tamoxifen in a dose-dependent manner. Error bars indicate SE. ^*^ indicates significant difference from DMSO 2.5%. One-Way ANOVA P=0.004, Tukey-Kramer post-hoc P<0.05. a indicates significantly different from Tamoxifen 0 µg/ml at respective C10 Dosages. One-Way ANOVA P=0.002, P=0.0001, P=0.00001, for C10 0mM, 0.25 mM, and 0.5 mM, respectively. Tukey-Kramer post-hoc P < 0.01 for all comparisons. Bars indicate significant differences as indicated. One-Way ANOVA P=0.003. Tukey-Kramer post-hoc P<0.05. Results presented are representative of at least n = 3 separate experiments.

Tamoxifen is the current standard of care therapy for all stages of breast cancer; however, resistance to the drug has been a major concern [29]. The development of drugs that can be used in combination with tamoxifen may help to increase its effectiveness in treating breast cancer. Since C10 decreased tumor cell viability/growth of other types of human cancer cells, we then sought to determine if C10 could work alone or synergistically in combination with tamoxifen to further inhibit tumor cell viability/growth. As shown in Figure 4, treatment with C10 resulted in significantly reduced MCF-7 cell viability/growth. Moreover, C10 in combination with tamoxifen resulted in a more robust inhibitory effect of tamoxifen on cell viability/growth of MCF-7 cells (Figure 4). These results indicate that the combination of a pathologic TLR3 inhibitor (e.g. C10) and tamoxifen could potentially be of interest for treating breast cancer.

## DISCUSSION

A significant number of women across different geographies are afflicted with breast cancer. Estrogen plays a critical role in pathology of the vast majority of these breast cancers. Accordingly, patients are treated with anti-estrogen therapies (e.g., tamoxifen). However, these anti-estrogen therapies have their limitations: (i) some patients have inadequate response to these therapies and (ii) patients can develop resistance to these therapies – eventually relapsing. Hence, there remains a significant unmet medical need to develop better and novel therapeutics (monotherapy or combination therapy) for this common disease.

Recent studies have highlighted the important pathologic role of TLR3 and Wnt5a in progression, sustenance and metastasis of breast cancer [30, 31]. Our group has identified phenylmethimazole (C10), an inhibitor of pathologic TLR3 signaling/expression, and shown that C10, by virtue of inhibiting the TLR3/Wnt5a pathway is significantly efficacious in *in-vitro* and *in-vivo* models of different cancers and auto-immune diseases [1, 2, 32, 33]. Accordingly, in this study, we sought to further investigate the effects of C10 on human breast tumor cells alone and in combination with standard of care therapy, tamoxifen. Our results demonstrate that C10 inhibits TLR3 and Wnt5a mRNA expression in MCF-7 breast cancer cells. Subsequently, C10 significantly reduced IL-6 protein levels as well as downstream STAT3^Y705^ phosphorylation. Functionally, the inhibition of this signaling pathway translates into the inhibition of viability/growth and migration of the MCF-7 breast cancer cells. These observations are in-line with our previous studies examining the inhibition of TLR3 signaling with C10 in various other TLR-expressing cancers and the importance of TLR3 signaling modulation as a potential therapeutic target for breast cancer [1, 2, 34].

Although this current study, as well as previously published data, revealed viability/growth inhibition of various tumor cell lines with a TLR signaling inhibitor, we furthered these experiments to investigate the potental combinational use of a TLR signaling inhibitior with a currently FDA-approved drug used to treat breast cancer, tamoxifen. Strikingly, we observed that C10 in combination with tamoxifen synergistically enhanced the cytotoxic potential of tamoxifen by more than 50% when compared to tamoxifen therapy alone. These data suggest that a TLR3 inhibitor alongside tamoxifen, and potentially other existing ER therapies, may be a potential novel therapeutic approach to enhance current standard of care therapies. Future studies need to be conducted *in vivo* to determine if this combination could be applicable in the clinic.

## MATERIALS AND METHODS

### Cells

Human breast cancer cell line MCF-7 was purchased from ATCC (Manassas, VA). Cells were grown in RPMI 1640 supplemented with 2 g/L sodium bicarbonate, 1.4 mmol/L sodium pyruvate, 0.14 mmol/L nonessential amino acids, and 10% fetal bovine serum (pH 7.2). Phenylmethimazole (C10 [1, 2, 32, 33]) was prepared as 200 mM stock solution in DMSO and diluted in cell culture media.

### Real-Time PCR

Total RNA was isolated (RNeasy Kit, Qiagen, Valencia, CA, USA) and treated with DNase (RNase-Free DNAse Kit, Qiagen). cDNA was synthesized using the High Capacity cDNA Reverse Transcription Kit (Applied Biosystems, Carlsbad, CA, USA). Pre-amplification of cDNA was done using the TaqMan PreAmp Master Mix Kit (Applied Biosystems, Carlsbad, CA, USA). Multiplex real-time PCR was performed using human TLR3 and Wnt5a Gene Expression Assays and GAPDH Gene Expression Assay was used as the internal control (Applied Biosystems). Non-preamplified cDNA was used for Wnt5a real-time PCR, whereas pre-amplified cDNA was used to amplify TLR3 by real-time PCR. Changes in gene expression were calculated using the ΔΔCt method.

### ELISAs

Human recombinant IL-6 and pSTAT3^Y705^ ELISA kits were from Life Technologies (Grand Island, NY). For the measurement of IL-6, cells were treated for 24 hours with 500 μM C10 or DMSO (0.25%) for 24 hours. Supernatants were collected and IL-6 levels were evaluated using the human IL-6 ELISA kit as per the manufacturer’s instructions. For detection of pSTAT3^Y705^, cells were pre-treated with 500 μM C10 or DMSO (0.25%) for 1 hour and then stimulated with 100 ng/mL human recombinant IL-6 for ten minutes. Nuclear proteins were then collected using the NE-PER kit from ThermoFisher Scientific (Waltham, MA) as per the manufacturer’s instructions. Nuclear protein was then used to determine pSTAT3^Y705^ levels via the pSTAT3^Y705^ ELISA kit as per the manufacturer’s protocol.

### Cell viability/growth

MCF-7 cells were evenly seeded and grown on sterile 96-well plates. Cells were then treated with C10 (0, 250, or 500 μM), DMSO (0.25%), or tamoxifen (6 μg/mL; Sigma Aldrich) alone or in combination for 72 hours. Cell viability/growth was then quantified using the MTT-based *In Vitro* Toxicology Assay Kit from Sigma-Aldrich (St. Louis, MO).

### Cell migration

Scratch assays were performed as previously described [1, 2]. Briefly, MCF-7 cells were grown to confluency and a scratch was made in the confluent layer of cells using a sterile pipette tip. In some instances the cells were treated with C10 (500 μM) or 0.25% DMSO. Scratch assays were documented after 24 and 48 hrs using digital photography. ImageJ (NIH) was used to analyze 3 separate images of each treatment group to quantify cellular migration.

### Statistics

All experiments were replicated at least three times on different groups of cells. All data are expressed as mean ± SD. Statistical significance was evaluated using a one-way ANOVA, and statistical significance for comparison of means between different groups was calculated using the Tukey-Kramer multiple comparison post hoc analysis using NCSS software. The differences were considered significant at p values < 0.05 as indicated in the figure legends.
